# Gene Selection for Multiclass Prediction by Weighted Fisher Criterion

**DOI:** 10.1155/2007/64628

**Published:** 2007-07-10

**Authors:** Jianhua Xuan, Yue Wang, Yibin Dong, Yuanjian Feng, Bin Wang, Javed Khan, Maria Bakay, Zuyi Wang, Lauren Pachman, Sara Winokur, Yi-Wen Chen, Robert Clarke, Eric Hoffman

**Affiliations:** 1 Department of Electrical and Computer Engineering, Virginia Polytechnic Institute and State University, Arlington, VA 22203, USA; 2 Department of Pediatric Oncology, National Cancer Institute, Gaithersburg, MD 20877, USA; 3 Research Center for Genetic Medicine, Children's National Medical Center, Washington, DC 20010, USA; 4 Disease Pathogenesis Program, Children's Memorial Research Center, Chicago, IL 60614, USA; 5 Department of Biological Chemistry, University of California, Irvine, CA 92697, USA; 6 Lombardi Cancer Center, Georgetown University, Washington, DC 20007, USA

## Abstract

Gene expression profiling has been widely used to study molecular signatures of many diseases and to develop molecular diagnostics for disease prediction. Gene selection, as an important step for improved diagnostics, screens tens of thousands of genes and identifies a small subset that discriminates between disease types. A two-step gene selection method is proposed to identify informative gene subsets for accurate classification of multiclass phenotypes. In the first step, individually discriminatory genes (IDGs) are identified by using one-dimensional weighted Fisher criterion (wFC). In the second step, jointly discriminatory genes (JDGs) are selected by sequential search methods, based on their joint class separability measured by multidimensional weighted Fisher criterion (wFC). The performance of the selected gene subsets for multiclass prediction is evaluated by artificial neural networks (ANNs) and/or support vector machines (SVMs). By applying the proposed IDG/JDG approach to two microarray studies, that is, small round blue cell tumors (SRBCTs) and muscular dystrophies (MDs), we successfully identified a much smaller yet efficient set of JDGs for diagnosing SRBCTs and MDs with high prediction accuracies (96.9% for SRBCTs and 92.3% for MDs, resp.). These experimental results demonstrated that the two-step gene selection method is able to identify a subset of highly discriminative genes for improved multiclass prediction.

## 

[1,2,3,4,5,6,7,8,9,10,11,12,13,14,15,16,17,18,19,20,21,22,23,24,25,26,27,28,29,30,31,32,33,34,35,36,37,38,39,40,41,42,43,44,45,46,47]
